# Daily variation in the electrophysiological activity of mouse medial habenula neurones

**DOI:** 10.1113/jphysiol.2013.263319

**Published:** 2013-12-16

**Authors:** Kanwal Sakhi, Mino D C Belle, Nicole Gossan, Philippe Delagrange, Hugh D Piggins

**Affiliations:** 1 Faculty of Life Sciences, University of ManchesterManchester, UK; 2 Unité de Recherches et Découvertes en Neurosciences, Institut de Recherches Servier78290 Croissy-sur-Seine, France

## Abstract

**Abstract**Intrinsic daily or circadian rhythms arise through the outputs of the master circadian clock in the brain's suprachiasmatic nuclei (SCN) as well as circadian oscillators in other brain sites and peripheral tissues. SCN neurones contain an intracellular molecular clock that drives these neurones to exhibit pronounced day–night differences in their electrical properties. The epithalamic medial habenula (MHb) expresses clock genes, but little is known about the bioelectric properties of mouse MHb neurones and their potential circadian characteristics. Therefore, in this study we used a brain slice preparation containing the MHb to determine the basic electrical properties of mouse MHb neurones with whole-cell patch clamp electrophysiology, and investigated whether these vary across the day–night cycle. MHb neurones (*n* = 230) showed heterogeneity in electrophysiological state, ranging from highly depolarised cells (∼ −25 to −30 mV) that are silent with no membrane activity or display depolarised low-amplitude membrane oscillations, to neurones that were moderately hyperpolarised (∼40 mV) and spontaneously discharging action potentials. These electrical states were largely intrinsically regulated and were influenced by the activation of small-conductance calcium-activated potassium channels. When considered as one population, MHb neurones showed significant circadian variation in their spontaneous firing rate and resting membrane potential. However, in recordings of MHb neurones from mice lacking the core molecular circadian clock, these temporal differences in MHb activity were absent, indicating that circadian clock signals actively regulate the timing of MHb neuronal states. These observations add to the extracellularly recorded rhythms seen in other brain areas and establish that circadian mechanisms can influence the membrane properties of neurones in extra-SCN sites. Collectively, the results of this study indicate that the MHb may function as an intrinsic secondary circadian oscillator in the brain, which can shape daily information flow in key brain processes, such as reward and addiction.

## Introduction

Daily near-24 h or circadian rhythms in physiology and behaviour emerge through the activities of intrinsic circadian oscillators in the brain and body and their synchronisation (entrainment) to recurrent environment signals, including variation in environmental lighting, food availability and social interactions (Dibner *et al*. 2010; Piggins & Guilding, 2011; Bechtold & Loudon, 2013). The brain's suprachiasmatic nuclei (SCN) house the dominant light-entrainable circadian clock, and many SCN neurones contain an intracellular molecular clock of which the *Period* (*Per1–2*) and *Cryptochrome* (*Cry1–2*) genes and their protein products (PER1–2, CRY1–2) are important constituents (Ko & Takahashi, 2006; Welsh *et al*. 2010). This intracellular transcription–translation feedback loop completes a cycle in ∼24 h and drives SCN neurones to show pronounced day–night differences in their electrical activity (Brown & Piggins, 2007; Colwell, 2011). This facilitates the conveying of circadian signals from the SCN to the rest of the brain and body. Remarkably, such circadian changes in SCN neuronal activity and clock gene expression are readily measured in SCN brain slices and are absent in the SCN of adult rodents lacking the molecular oscillator (e.g. *Cry1^−/−^Cry2^−/−^* mice (Albus *et al*. 2002; Ono *et al*. 2013).

The SCN was the first neural pacemaker to be identified in mammals, but evidence over the past 15 years establishes that other neural sites and peripheral tissues rhythmically express circadian clock genes (Guilding & Piggins, 2007; Dibner *et al*. 2010). One such brain structure is the habenula (Hb) which is located above the dorsal thalamus (Herkenham & Nauta, 1979; Kim, 2009). Functionally, the Hb acts as a relay station, conveying neural information from the forebrain to mid- and hind-brain structures (Sutherland, 1982; Hikosaka, 2010). Anatomically, this epithalamic region is positioned adjacent to the dorsal third ventricle and is composed of two major components, the medial (MHb) and the lateral (LHb) habenula, respectively. Current evidence implicates the MHb in nicotine addiction (Fowler *et al*. 2011; Stoker & Markou, 2013), cognition and memory (Lecourtier *et al*. 2004; Sanders *et al*. 2010; Kobayashi *et al*. 2013), sleep (Goldstein, 1983; Haun *et al*. 1992) and fear and anxiety (Jesuthasan, 2011; Yamaguchi *et al*. 2013). Circadian clock gene expression is reported in the MHb (Yamamoto *et al*. 2001; Shieh, 2003), and light input pathways that regulate SCN activity also innervate the habenula (Hattar *et al*. 2006) and appear functional since retinal illumination can influence MHb electrical activity (Zhao & Rusak, 2005). Moreover, gene expression in the MHb can be regulated by SCN-derived signals (Yu *et al*. 2002). Intriguingly, using a mouse in which luciferase (Luc) reports the production of PER2, weak circadian rhythms in PER2::Luc bioluminescence can be visualised in the ependymal cells that border the MHb (Guilding *et al*. 2010). Collectively, these studies raise the possibility that circadian signals influence MHb neuronal activity.

Although there is considerable knowledge of the potential functions and anatomical connections of the MHb in rodent models, much less is known about the electrophysiological properties of MHb neurones, particularly in mouse. In this study, we present the first comprehensive examination of bioelectrical properties in the mouse MHb neurones *in vitro*. We report that, when isolated from the SCN, mouse MHb neurones show four main states of spontaneous activity, ranging from highly depolarised, non-firing (silent) cells to neurones resting at moderate resting potentials and generating action potentials. We show that there is significant variation in the resting membrane potential of MHb neurones across the day–night cycle, and that the spontaneous firing rate is significantly lower in the morning compared to the late day and early night. We find that immunostaining for the small conductance calcium-activated potassium channel (SK) is dense in the MHb, and that pharmacological activation of this conductance reversibly leads to hyperpolarisation of MHb neurones. This indicates that variation in SK channel activity contributes to MHb neuronal states. Temporal variation in the electrophysiological properties of MHb neurones is absent in recordings from mice lacking the intracellular molecular clock. Circadian oscillations in a bioluminescent reporter of the molecular clock are present in habenula brain slices, but absent in slices from mice lacking the intracellular molecular clock. These studies demonstrate that intrinsic and/or extrinsic circadian signals influence MHb neuronal activity states.

## Methods

### Animals

Mice in which a destabilised enhanced green fluorescent protein (EGFP) reports the expression of a clock gene, *Per1* (*Per1*::d2EGFP mice, initial breeding stock provided by Professor D. McMahon, Vanderbilt University, USA) were bred in the Biological Services Facility at the University of Manchester. Animals were bred under a 12 h:12 h light–dark (LD) cycle. Once weaned, mice were group housed under 12 h:12 h LD conditions (with Zeitgeber (ZT) 0 defined as lights-on) until use in experiments. A total of 89 male and female *Per1*::d2EGFP mice (aged 4 weeks to 4 months) were used. Preliminary investigations indicated no obvious sex difference in MHb neuronal activity and so the MHb recordings from male and female mice were combined. Some of the SCN tissue from these mice was used for other studies (Scott *et al*. 2010; Diekman *et al*. 2013) and to ethically reduce the number of animals, we used the MHb slices for our studies. Since one aim of this study was to investigate possible circadian variation in the basic electrical properties of MHb neurones, for convenience we used animals housed in three different sets of LD cycles. To record between ZT 5 and 14 we used mice kept on a 07.00–19.00 h LD cycle with lights-on at 07.00 h and lights-off at 19.00 h. Recordings between ZT 14 and 22 were made using mice housed in a reversed LD cycle (lights-on: 23.00–11.00 h), and finally for recording between ZT 22 and 5 animals were kept in a delayed LD cycle (lights-on: 15.00–03.00 h). To further examine the contribution of the conventional molecular circadian clock to the electrophysiological properties of MHb neurones, we also used mice deficient in the *Cryptochrome* genes. *Cry1^+/−^Cry2^+/−^* animals (van der Horst *et al*. 1999) that had been bred with *Per1*-luc mice (Yamaguchi *et al*. 2000) carrying a *Per1* luciferase reporter were obtained from Erasmus Medical Centre, Rotterdam, The Netherlands. These mice had been backcrossed with C57BL/6J mice for nine generations. From *Cry1^+/−^Cry2^+/−^* × *Per1*-luc breeding pairs, we genotyped offspring and used adult (2–6 months of age) animals lacking the molecular clock (referred to here as *Cry1^−/−^Cry2^−/−^* mice) as well as congenic littermates in which the molecular clock is fully functional (referred to here as *Cry1^+/+^Cry2^+/+^* mice). Prior to use in electrophysiology experiments, these animals were housed on a 12 h:12 h LD cycle (either lights-on at 07.00 h or lights-on at 23.00 h) for a minimum of 4 weeks. To reduce the potential masking influence of the LD cycle, the mice were transferred into constant dark (DD) for 3 days prior to use in experiments. *Cry1^−/−^Cry2^−/−^* mice are behaviourally arrhythmic, but since *Cry1^+/+^Cry2^+/+^* mice have an intrinsic rhythm of ∼23.8 h (M. D. C. Belle & H. D. Piggins, unpublished observations), we projected this to predict ZT 12 (which is the onset of the circadian subjective night) and prepared brain slices from mice during the subjective day. For bioluminescence experiments, four *Cry1*^+/+^*Cry2*^+/+^ and four *Cry1^−/−^Cry2^−/−^* mice were group housed, maintained under a 12 h:12 h LD cycle, and killed (as described below for brain slice preparation for in vitro electrophysiology) at ∼ZT 3–4. Food (Bekay, B&K Universal, Hull, UK) and water was provided *ad libitum*. All animal protocols were in accordance with guidelines of the UK Animal (Scientific Procedure) Act 1986.

### Brain slice preparation for *in vitro* electrophysiology

Mice were deeply anaesthetised by inhalation of isoflurane (Abbott Laboratories, Kent, UK) and killed by decapitation. The brain was quickly removed and immersed in cold, oxygenated (95% O_2_; 5% CO_2_), low Na^+^/Ca^2+^, high Mg^2+^, sucrose-based incubation artificial cerebrospinal fluid (aCSF). This incubation aCSF contained (in mm): NaCl 95, KCl 1.8, KH_2_PO_4_ 1.2, CaCl_2_ 0.5, MgSO_4_ 7, NaHCO_3_ 26, glucose 15, sucrose 50; Phenol Red was added at 0.005 mg l^−l^; pH was 7.4 and measured osmolality was 300–310 mosmol kg^−l^. A coronal block of tissue (∼0.5 cm thick) containing the habenula was isolated, mounted on a stage, and coronal slices of 200–250 *μ*m thickness (corresponding to the region between ∼1.34 and ∼1.46 mm from bregma; Paxinos & Franklin, 2001) were cut using a vibroslicer (Campden Instruments, Leicester, UK). Slices were prepared during ZT1–10. For animals housed in DD, animal handling and brain extraction were performed with the aid of night vision goggles to prevent exposure of animals to visible light.

Individual slices containing the intermediate level of the habenula (along the rostro-caudal axis) were then directly transferred to a recording chamber mounted on the stage of a microscope and continuously perfused (∼2 ml min^−l^) with recording aCSF. The ionic composition of the recording aCSF was (in mm): NaCl 127, KCl 1.8, KH_2_PO_4_ 1.2, CaCl_2_ 2.4, MgSO_4_ 1.3, NaHCO_3_ 26, glucose 15; Phenol Red was added at 0.005 mg l^−l^; pH was 7.4 and measured osmolality was 300–310 mosmol kg^−l^; oxygenated with 95% O_2_–5% CO_2_. Slices were incubated in recording aCSF for at least 1.5 h prior to commencement of electrophysiological recordings.

### Whole-cell recordings

Patch electrodes were pulled from thick-walled borosilicate glass capillaries (Harvard Apparatus Ltd, Kent, UK) with a two-stage vertical micropipette puller (PB-7, Narishige, Tokyo, Japan). Resistance of the electrodes was 7–10 MΩ, and they were filled with an intracellular solution containing (in mm): potassium gluconate 130, KCl 10, MgCl_2_ 2, K_2_-ATP 2, Na-GTP 0.5, Hepes 20, EGTA 0.5. The pH was adjusted to 7.28 with KOH and measured osmolality was 295–300 mosmol kg^−l^.

Initially, a ×10 objective was used to identify the MHb, and subsequently the MHb neurones were visualised on a video screen using an Olympus BX51W1 microscope (Olympus UK, Southend-on-Sea, UK), equipped with infra-red video-enhanced differential interference contrast (IR/DIC) optics. Under microscopic examination, the MHb was readily distinguished from the adjacent dorsal third ventricle and floor of the lateral ventricles. The microscope also incorporated filters optimised for visualising green fluorescent protein under a ×40 water immersion objective. To identify the *Per1*::EGFP-expressing habenula neurones, a fast integrating highly sensitive camera system (ORCA R2 cooled digital charged-coupled device camera, Hamamatsu UK, Hertfordshire, UK) coupled with capturing software (HCImage Software and Drivers, Hamamatsu) was used. When targeting the neurones containing EGFP, care was taken to only briefly illuminate the slices with the fluorescence light.

Cell membrane was ruptured under minimal holding currents and negative pressure. All data were collected in current-clamp mode using a BA-O3X bridge amplifier (npi electronic GmbH, Tamm, Germany). Access resistance for all cells used in the analysis was ∼15 MΩ. Series resistance was ∼20 MΩ, and cells were discarded from the analysis if under basal conditions this value changed by more than 15%. Neurones were also removed from analysis if their resting membrane potential (RMP) was unstable after membrane rupture in whole-cell configuration. Signals were sampled at 30 kHz, stored and analysed on a computer using spike2 software (version 6.00: Cambridge Electronic Design (CED), Cambridge, UK). All data acquisition and stimulating protocols were generated through a micro1401 mkII interface (CED). Neurones, including the EGFP-positive cells, were recorded throughout the MHb. To confirm the accurate targeting of EGFP-expressing habenula neurones, patch pipettes sealed to these neurones were photographed *in situ* (×40 objective) at the end of each recording. All MHb neurone–patch pipette assemblies were also photographed at ×10 magnification to verify their anatomical localisation within this brain structure.

### Membrane properties of MHb neurones

Once the whole-cell configuration was established, neurones were allowed to recover for at least 1 min. Membrane properties, such as resting membrane potential (RMP), spontaneous firing rate and input resistance (*R*_input_) were determined within 4–5 min of membrane rupture to minimise any potential washout effects from whole-cell recordings. RMP was measured as follows: after ∼1 min when the RMP was stable, a 10 s trace was selected and two cursors were placed on each side. Subsequently, a custom-written Spike2 script was used to estimate the RMP of this trace marked by these two cursors. To determine the action potential (AP)/AP-like spike/membrane oscillation amplitude, we quantified the difference between peak voltage and the RMP of the spike, whereas the spike width was measured at half-amplitude (see Supplemental Fig. S1A, available online, and Pennartz *et al*. 1998). Further, *R*_input_ was estimated using Ohm's law (*R* = *V*/*I*), where *V* is calculated from the instantaneous voltage deflection induced by negative current pulses (−20 pA; 500 ms) (Supplemental Fig. S1B). The neurone's response to excitatory and inhibitory stimuli was identified by a series of depolarising (10–30 pA; 1 s duration) and hyperpolarising (−10 to −30 pA; 500 ms duration) current injections. This was then used to appropriately categorise the cells. In some cells, the termination of the hyperpolarising current injection (−20 pA; 500 ms) resulted in a rebound spike that was immediately followed by an after-hyperpolarisation (AHP). The amplitude of this AHP was measured between the trough of the AHP and the RMP of the cell before the current injection. The AHP duration was taken between the onset of the AHP and the time point where the membrane potential returns to the baseline value (Supplemental Fig. S1C).

Generally, recordings were made for approximately 10 min from each cell. However, cells receiving tetrodotoxin (TTX) and/or NS309 applications were recorded for up to 1 h. Since whole-cell patch recording cannot be used to record from a single cell over the entire circadian cycle, we recorded from groups of neurones (∼5 neurones per animal) at various circadian phases and compared their electrical characteristics as previously described (see Belle *et al*. 2009).

### Immunohistochemistry

Six adult mice (3 male and 3 female) were terminally anaesthetised with pentobarbital (80/mg/kg; ip) between ZT 8 and 12 and transcardially perfused with Kreb's solution, followed by 4% paraformaldehyde in PBS. Brains were removed, post-fixed in 4% paraformaldehyde for 24 h and then transferred to 30% sucrose solution for 3 days for cryoprotection. Whole brains were then flash-frozen in crushed dry ice and sectioned at 40 *μ*m using a freezing sledge microtome (Bright Instruments, UK). Immunohistochemistry was performed on free-floating brain sections as previously described (Marston *et al*. 2008) using a rabbit polyclonal antibody against the small-conductance calcium-activated potassium channel K_Ca_2.3 (SK3) (Alomone Labs; Jerusalem, Israel) at a concentration of 1:1000, and a donkey anti-rabbit biotinylated secondary antibody at 1:500 (Jackson Immunoresearch, Stratech Scientific Ltd, Suffolk, UK). Enzymatic detection and staining was performed using a nickel-intensified diaminobenzidine chromagen (NiDAB), catalysed by 0.015% glucose oxidase (Sigma-Aldrich, Poole, UK). Sections were then mounted, coverslipped and imaged using an Olympus BX-50 microscope and an attached Olympus c-4000-z digital camera. Control experiments omitted primary antibody.

### *Per1*-luc bioluminescence

#### Culture preparation

Mice on the *Per1*-luc background were anaesthetised by inhalation of isoflurane (Abott Laboratories) and killed by cervical dislocation. Brains were immediately removed and moistened with ice-cold Hanks’ balanced salt solution (HBSS; Sigma, UK) supplemented with 0.035% sodium bicarbonate (Sigma), 0.01 m Hepes (Sigma) and 1 mg ml^−l^ penicillin–streptomycin (Gibco Invitrogen Ltd, Paisley, UK). A coronal block containing the habenula was isolated and 300 *μ*m-thick brain slices were cut using a vibroslicer (Camden Instruments) in cold HBSS. Slices were then transferred to sterile tissue culture dishes (Corning Inc., Corning, NY, USA), and using a dissecting microscope the bilateral habenular complex was microdissected (as described in Guilding *et al*. 2009, 2010). Excised tissue was cultured on interface-style Millicell culture inserts (PICMORG50, Millipore (UK) Ltd, Watford, UK) in standard 35 mm plastic-based cultures dishes (Corning, UK). Each dish contained 1 ml of sterile culture medium (Dulbecco's modified Eagle's medium; DMEM (D-2902, Sigma)) supplemented with 3.5 g l^–1^ d–glucose (Sigma), 0.035% sodium bicarbonate (Sigma), 10 mm Hepes buffer (Sigma), 1 mg ml^−l^ penicillin–streptomycin (Gibco); B27 (Invitrogen) or 5% fetal bovine serum (FBS; Gibco) and 0.1 mm luciferin (Promega, Southampton, UK) in autoclaved (Milli-Q water). Dishes were sealed with a glass coverslip using autoclaved high-vacuum grease (Dow Corning Ltd, Coventry, UK) and transferred directly to the photomultiplier tube incubators for bioluminescence recording.

#### Luminometry

Total bioluminescence was recorded for up to 5 days from individual brain slice cultures with photomultiplier tube assemblies (H8259/R7518P, Hamamatsu, Welwyn Garden City, UK) housed in a light tight incubator (Galaxy R+, RS Biotech, Irvine, UK) maintained at 37°C. Photon counts were integrated for 299 s every 300 min.

#### Bioluminescence data analysis

Bioluminescence data were detrended by subtracting a 24 h running average from the raw data and smoothed with a 3 h running average. The resulting columns of data were plotted and period (peak–peak and trough–trough averaged) and rate of damping (the number of cycles observed before bioluminescence levels reached the previously determined level of dark noise (±10%)) were assessed manually by two experienced, independent researchers.

### Drugs and statistical analysis

Tetrodotoxin (TTX) was obtained from Tocris (Avonmouth, Bristol, UK), and 6,7-dichloro-1*H*- indole-2,3-dione 3-oxime (NS309) from Sigma. NS309 was initially dissolved in dimethyl sulphoxide (DMSO) (Sigma) and during the experiments stock solutions were diluted in recording aCSF and bath applied in the perfusing aCSF. The final concentration of DMSO was <0.001%. Most data were statistically analysed using Graphpad Prism Version 5.04 (Graphpad, La Jolla, CA, USA), while some data were analysed using Systat Version 10 (Systat Software Inc., Chicago, IL, USA). Most grouped data were analysed by one-way ANOVA followed by Tukey's *post hoc* test or paired *t* test, while some were analysed by two-way ANOVA followed by *a priori* single degree of freedom tests. Values were considered statistically significant if *P* < 0.05. All values, both in the text and in graphs, are presented as mean ± SEM.

## Results

In this study, successful whole-cell patch-clamp recordings were performed across the 24 h of the LD cycle from a total of 276 MHb neurones from *Per1*::GFP mice. Our original intention was to target EGFP+ve cells only, but unlike in SCN brain slices from the same mouse genotype, the level of expression of *Per1*-driven EGFP was too low to be useful, presumably due to the small (∼3 kb; Kuhlman *et al*. 2000) *Per1* promotor fragment used to drive the EGFP in these mice (see Supplemental Fig. S2) as well as reflecting the much lower expression of clock genes in the MHb as compared with the SCN (Yamamoto *et al*. 2001; Shieh, 2003). Indeed in pilot studies, we found no clear differences in the electrophysiological properties of EGFP+ve and –ve MHb cells (data not shown). Therefore, in all subsequent recordings, no attempt was made to differentiate and target EGFP+ve and –ve neurones in the MHb. Since this is the first investigation of MHb neurones in mice, we describe the various states of MHb neurones with respect to their membrane properties, and subsequently discuss the circadian aspect of these electrical properties.

### Four states of MHb neurones

To determine the basic membrane properties of mouse MHb neurones, we initially recorded from 93 cells across the middle to late day (ZT 8–12). These neurones showed heterogeneity in their basic electrical properties, particularly resting membrane potential (RMP). Based on their RMP, spontaneous electrical behaviour, and action potential (AP) amplitude and duration, we distinguished four basic states of MHb neurones: spontaneously AP-firing neurones, cells displaying depolarised low-amplitude membrane oscillations (DLAMO), non-spiking depolarised silent cells, and intermediate state cells showing a mixture of DLAMO and AP-like spikes. Consistent with earlier work in the rat (Kim & Chang, 2005), we found no obvious anatomical localisation of the different states within the MHb. Significant state-related variation in RMP as well as oscillation amplitude and duration were detected with one-way ANOVAs (all *P* < 0.01; see Fig. 1*E*–*G*).

**Figure 1:**
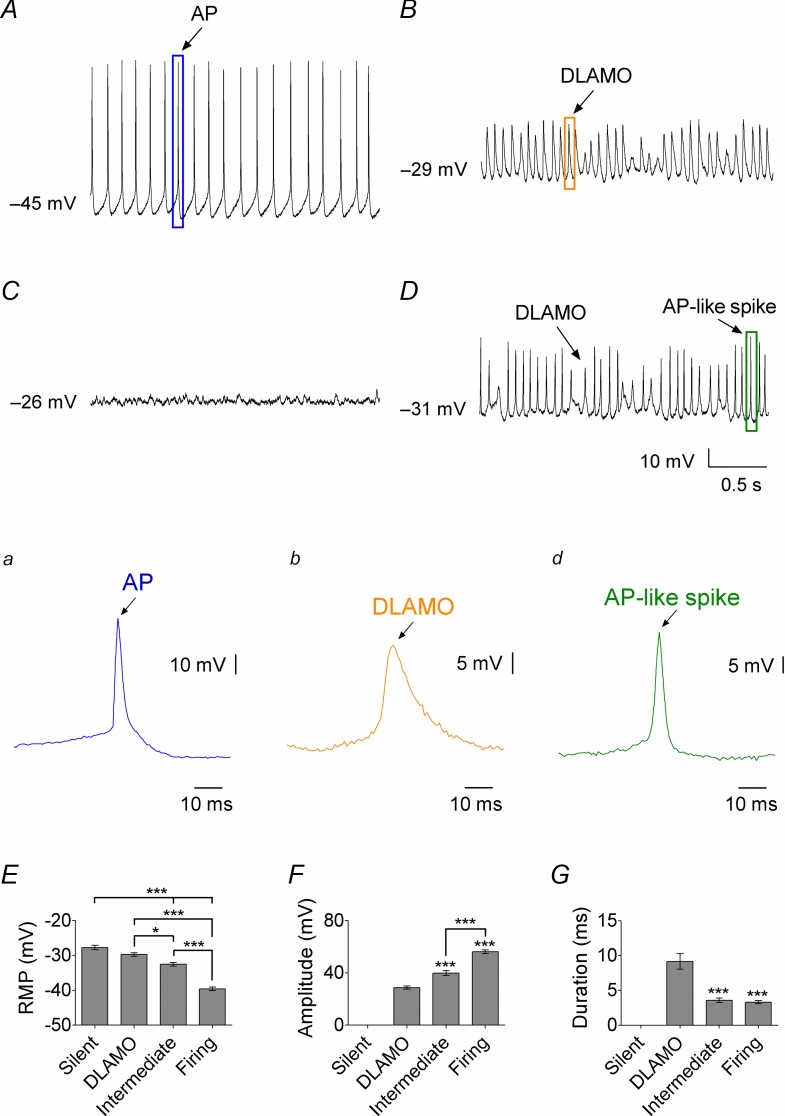
*A*, in one state, cells rest at a moderate RMP and generate tonic firing of APs. *B*, in another state, MHb neurones display DLAMOs. *C*, in a third state, MHb cells are mostly highly depolarised and silent. *D*, in a fourth intermediate state, MHb neurones switch between firing AP-like spikes and DLAMOs. Insets: enlarged AP (*a*), DLAMO (*b*) and AP-like spike (*d*). Note the different vertical scales. The AP-like spikes are significantly different from APs in amplitude (*P* < 0.001), but not in width (*P* > 0.05). The oscillations of the DLAMO cells have significantly shorter amplitude and a longer duration than other neuronal states (*P* < 0.0001). *E*, the mean RMP is significantly different in the four electrical states of MHb neurones (*P* < 0.0001). The oscillations of the DLAMO cells have significantly lower amplitudes (*F*) and longer durations (*G*) than other neurone states. In *E*–*G*, the mean and SEM are plotted. Tukey's *post hoc* test; *P < 0.05, ***P < 0.001.

#### Firing state

Neurones in this state (*n* = 39/93; 42%) spontaneously generated tonic trains of APs at frequencies between 0.2 and 24 Hz, with a mean AP firing rate of 7.6 ± 0.9 Hz (Fig. 1*A*). In this firing state, cells had an average RMP value of −39.5 ± 0.5 mV (Fig. 1*E*), with the AP amplitude and width averaging 56.3 ± 1.4 mV and 3.3 ± 0.2 ms, respectively (Fig. 1, inset *a*). The mean input resistance (*R*_input_) of cells in this state was estimated to be 2.2 ± 0.1 GΩ.

To further characterise the active membrane properties of MHb neurones in the firing state, a series of brief depolarising (10–30 pA, 1 s) or hyperpolarising pulses (−10 to −30 pA, 500 ms) were delivered to these cells (*n* = 39). In response to the depolarising pulse, the majority of these neurones (*n* = 26/39; 67%) showed frequency adaptation, such that they initially increased the firing of APs, but over the course of the pulse, the frequency and amplitude of the APs gradually decreased and the cells ceased firing APs (Fig. 2*A*). When given a 500 ms negative current injection, 17 (67%) of these cells produced a rebound AP immediately after the pulse was terminated (Fig. 2*E*), while 9 (33%) generated a delayed rebound AP that occurred >0.5 s following cessation of the negative current (Fig. 2*F*). The minority of firing MHb neurones tested (*n* = 13/39; 33%) did not show frequency adaptation to the depolarising pulse and instead maintained AP amplitude and elevated AP firing frequency throughout the depolarising stimulus (Fig. 2*B*). Most of these cells (9 of 13; 69%) also produced an immediate rebound spike after the brief hyperpolarising current was terminated, and four (33%) cells displayed a delayed rebound response to this hyperpolarising pulse. Following injection of the brief hyperpolarising current, some spontaneously firing neurones (*n* = 14/39; 36%) also showed a shallow (∼4 mV) after-hyperpolarisation (AHP), but most (*n* = 25/39; 64%) did not. Therefore in the firing state, MHb neurones demonstrate heterogeneity in their responses to depolarising and hyperpolarising stimuli.

**Figure 2:**
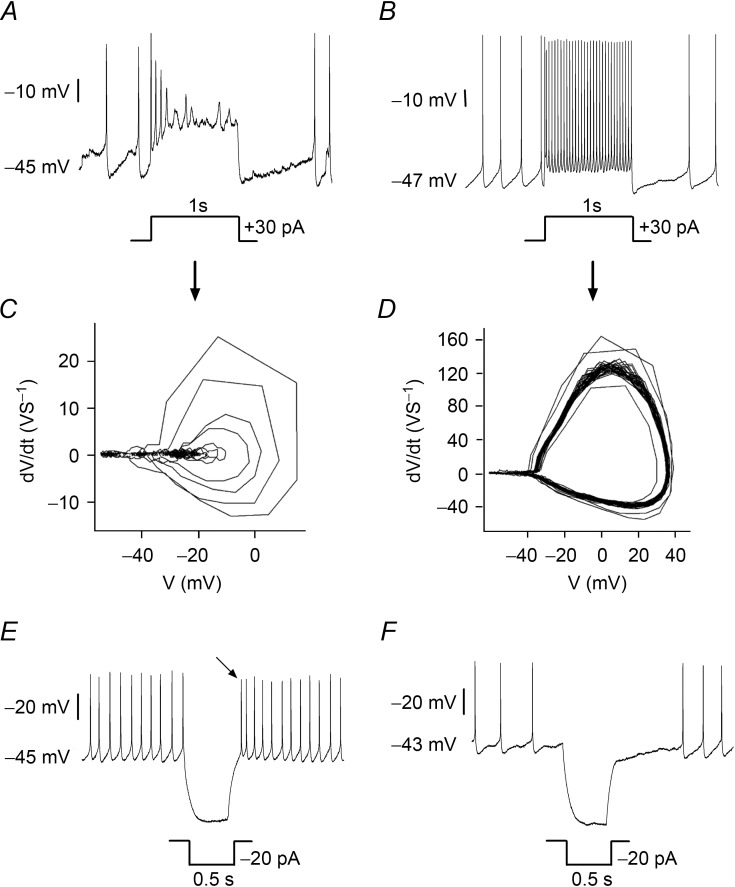
Most of the spontaneously firing MHb neurons (*n* = 26/39) responded to a depolarising current injection (30 pA; 1 s) with a rapid reduction in spike amplitude and overt firing frequency adaptation (*A*). However, in some MHb neurones (*n* = 13/39) that are spontaneously discharging APs, no reduction in AP amplitude or firing frequency adaptation was observed in response to a depolarising pulse (*B*). *C* and *D*, the resulting phase–plot curves show the AP velocity, trajectory and rate of frequency adaptation for these two groups of neurones. In MHb neurones, application of hyperpolarising pulses either produced a rebound spike in 17 cells (67%) (*E*, indicated by arrow), or resulted in a delayed response with no rebound spike (33%) (*F*).

#### Depolarised low-amplitude membrane oscillations (DLAMO) state

Besides the conventional firing state described above, we also observed MHb neurones spontaneously resting at more depolarised states. In these unusual states, MHb cells either displayed depolarised low-amplitude membrane oscillations (DLAMOs) (see Diekman *et al*. 2013) or were electrically silent (see ‘Silent state’ below and Belle *et al*. 2009). Cells in this DLAMO state (*n* = 28/93; 30%) were spontaneously depolarised and rested at membrane values between −25 and −34 mV (mean: −29.7 ± 0.5 mV) (Fig. 1*B*). These cells did not generate full APs but instead displayed DLAMOs with a mean frequency of 7.1 ± 1.1 Hz. The amplitude of these oscillations was significantly shorter than the APs measured in the firing state, and ranged between 18.7 and 41.1 mV (mean: −28.8 ± 1.2 mV; Tukey's *post hoc* test, *P* < 0.0001; Fig. 1, inset *b*), and the duration of individual oscillations was significantly longer than that of APs (mean: 9.2 ± 1.1 ms; Tukey's *post hoc* test, *P* < 0.0001; Fig. 1*G*). The mean *R*_input_ of cells in the DLAMO state was 2.0 ± 0.1 GΩ.

#### Silent state

Some depolarised MHb neurones (*n* = 16/93; 17%) completely lacked spontaneous discharge of APs or membrane oscillations and were electrically quiescent (Fig. 1*C*). These ‘silent’ cells were slightly more depolarised than neurones in the DLAMO states (mean: −27.7 ± 0.6 mV), and had an average *R*_input_ of 2.4 ± 0.2 GΩ.

#### Intermediate state

Finally, a number of MHb neurones (*n* = 10/93; 11%) resting between −30 and −34 mV (mean: −32.5 ± 0.5 mV) displayed a mixed behaviour, and therefore were ascribed to a fourth category called ‘intermediate state cells (Fig. 1*D*). Generally, neurones in this state switched their activity mode between DLAMOs and higher amplitude oscillations that partially resembled full APs. These AP-like spikes were significantly lower in amplitude (mean: 39.8 ± 1.9 mV) than APs measured in the firing cells, but higher in amplitude than DLAMOs (Tukey's *post hoc* test, *P* < 0.001; Fig. 1, inset *d*, and panel *F*). The width of these AP-like spikes (mean: 3.6 ± 0.3 ms), however, was similar to that of APs (Fig. 1, inset *d*, and panel *G*). The average *R*_input_ of these intermediate state neurones was 2.0 ± 0.1, and the frequency of these AP-like spikes was 7.7 ± 2.8 Hz.

To further characterise cells in the depolarised states, these neurones were given brief (0.5 s) hyperpolarising (−20 pA) pulses. In all the cells in the silent, DLAMO or intermediate states, the termination of this hyperpolarising pulse triggered an AHP accompanied by a train of APs. One such example is shown in Fig. 3*A*, where immediately following the −20 pA pulse, a previously silent cell displays an AHP as well as a train of APs. As the cell began to depolarise to the pre-pulse level, these APs gradually transitioned into DLAMOs and then the cell became electrically silent. Fig. 3*B* illustrates a similar response to a transient hyperpolarising pulse of a cell in the DLAMO state. The amplitude of this AHP varied according to the state of the neurone (one-way ANOVA, *P* < 0.05). The AHP amplitude was significantly larger in the silent (9.0 ± 1.2 mV) and DLAMO states (8.3 ± 0.6 mV), compared to cells in the firing states (5.9 ± 0.6 mV) (Tukey's *post hoc* test, *P* < 0.001; Fig. 3*C*), while the duration of the AHP did not significantly differ between the four MHb cell states (one-way ANOVA, *P* > 0.05; Fig. 3*D*). This indicates heterogeneity in MHb neuronal responses to a hyperpolarising stimulus and suggests that cell state could influence MHb neuronal processing of inhibitory inputs.

**Figure 3:**
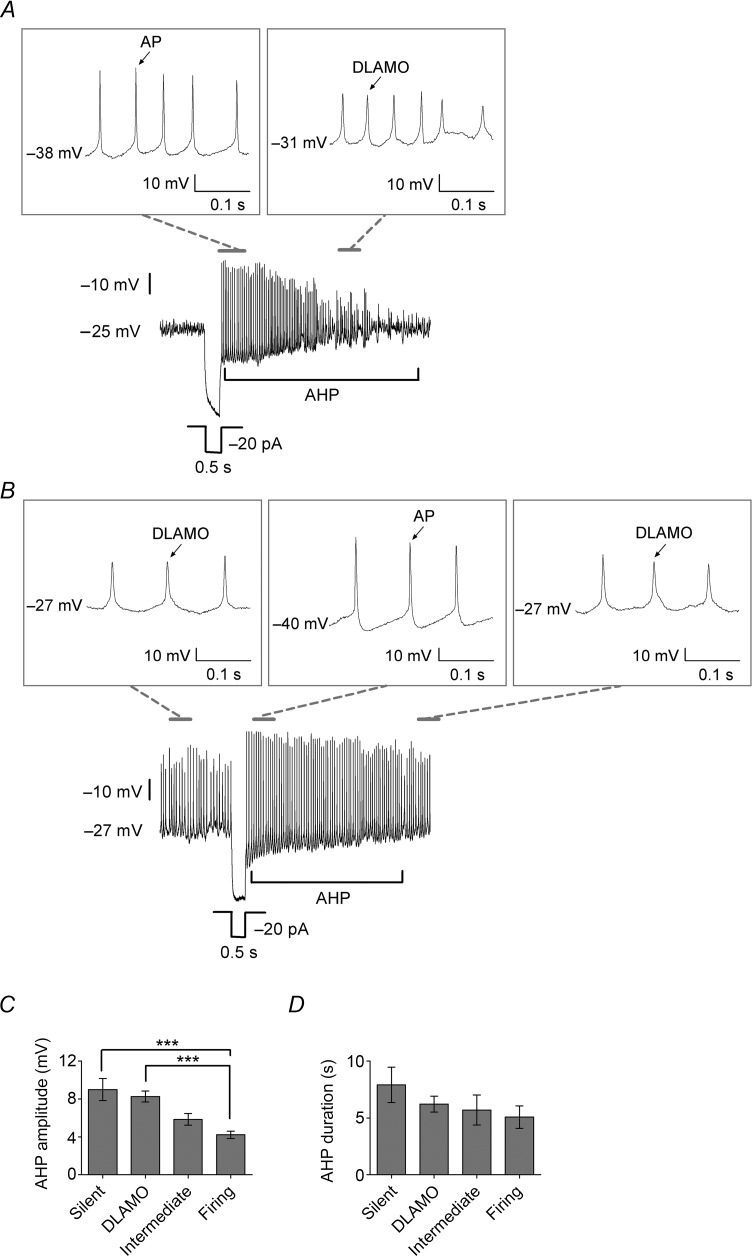
*A*, an example of a cell in the silent state where the termination of a brief hyperpolarisation pulse triggers an AHP that is accompanied by a train of APs. These APs gradually progress to oscillations in membrane potential while the RMP depolarises, and eventually the cell becomes silent again (see enlarged epochs in grey rectangles). *B*, a cell in the DLAMO state displaying spike train AHP upon termination of the hyperpolarising pulse. Enlarged epochs in the grey rectangles illustrate this progression. *C*, the AHP amplitude was significantly larger in the cells in the silent and DLAMO states, compared to that seen in cells in the firing state. *D*, the duration of the AHP did not differ significantly between the four MHb cell states. Tukey's *post hoc* test, ***P < 0.001.

### Transition of the various electrical states of MHb neurones

The observation that following hyperpolarising pulses, some MHb cells exhibit characteristics of the different spontaneous states of these neurones suggests that the states do not represent different types of neurones but rather display the continuum of possible MHb neuronal states. To test this, we sustained injections of positive or negative currents and determined if and how MHb neurones altered their spontaneous activity.

With application of the steady-state negative current injections, the neurones in the highly depolarised silent state (resting between −24 and −32 mV) changed to the DLAMO state (Fig. 4*A*). Further membrane hyperpolarisation to more negative values elicited firing of full APs in these previously silent cells. Likewise, for cells in the spontaneously firing state, manually sustaining them at more positive potential values, transitioned them to first display DLAMOs and subsequently they became completely silent (Fig. 4*B*). Interestingly, MHb cells in the firing state that did not show frequency adaptation to depolarising pulses (33%; *n* = 13/39) were unable to switch states in this way (data not shown). This indicates that the spontaneous electrical behaviour of many MHb neurones (67%) can switch to another pattern of activity as a function of their RMP. These observations strongly suggest that the various electrical states described are representative of a continuum of possible MHb neuronal states rather than separate neuronal types. The differential response of the firing cells to depolarising inputs does, however, suggest that there are at least two basic types of MHb neurones: adapting and non-adapting cells.

**Figure 4:**
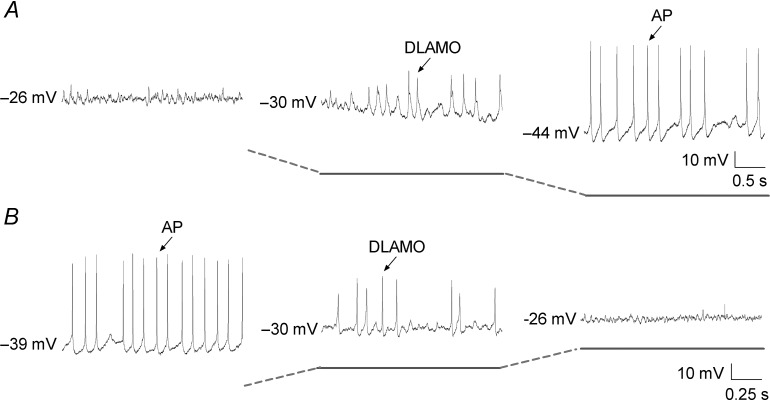
*A*, silent cells resting at highly depolarised states could be induced to display DLAMOs and APs by passing constant hyperpolarising current injections (horizontal grey line). *B*, likewise, MHb neurones that were spontaneously discharging APs moved to DLAMO state upon steady-state positive current injection, and eventually became completely silent.

The ionic mechanisms underpinning MHb neuronal states are unknown, but molecular and anatomical evidence indicates that mRNA for a member of the small-conductance calcium-activated potassium channels, SK3, is heavily expressed in this structure (Stocker & Pedarzani, 2000; Tacconi *et al*. 2001). Using antisera to SK3, we confirmed that anti-SK3 immunostaining is intense in the MHb, but sparse to absent in the adjacent LHb and dorsal thalamus (Fig. 5*A*). Such staining was absent when the primary antibody was omitted (Fig. 5*B*). To determine if activation of SK3 channels influences the electrical state of MHb neurones, we assessed the effects of NS309, a selective opener of SK channels (Strobaek *et al*. 2004), on MHb cells in the depolarised, silent and DLAMO states. In all the MHb neurones tested (*n* = 8), bath application of 20 *μ*m NS309 caused a 14.8 ± 1.2 mV hyperpolarisation of the membrane potential (paired *t* test, *P* < 0.01) (Fig. 5*C* and 5*D*). Variation was observed in the robustness of the NS309-induced responses which ranged from 8 to 33 mV, with maximal effects occurring 8.3 ± 1.8 min after the onset of application. Interestingly, the depolarised oscillations recorded under control conditions first transformed to full APs in the presence of NS309 (NS309 wash-in), and upon further hyperpolarisation (full NS309) these cells stopped producing APs and became completely silent (Fig. 5*C*). The NS309-induced hyperpolarisations appeared to occur through direct actions of NS309 on the postsynaptic cell, since they persisted in the presence of TTX (*n* = 5) (Fig. 5*D*). Following NS309 treatment, cells showed complete recovery within 30–40 min. These results suggest that alterations in SK channel activity contribute to changes in the states of MHb neurones.

**Figure 5:**
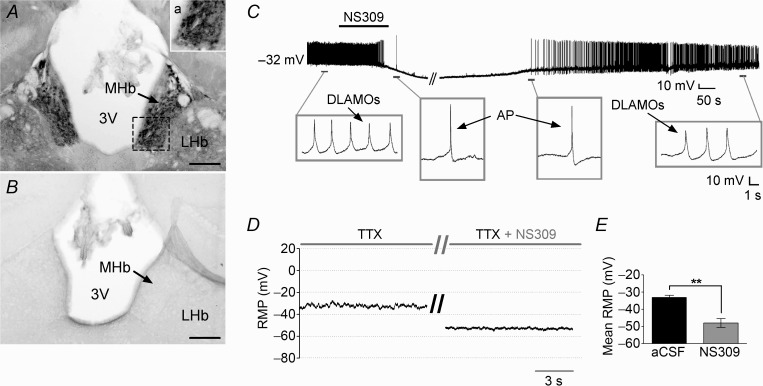
*A*, immunohistological localisation of the small-conductance calcium-activated potassium channel SK3 in the MHb of mouse brain. Inset *a*, a small area in *A* is enlarged showing SK3 staining in greater detail. *B*, no staining for SK3 channels is observed when primary antibody is omitted. *C*, application of 20 *μ*m NS309, an activator of small conductance Ca^2+^-activated K^+^ channels (SK1–3), hyperpolarised all the spontaneously depolarised silent and DLAMO-generating MHb neurones tested (*n* = 8). During the hyperpolarisation, the DLAMOs recorded under control conditions initially transformed into full APs upon NS309 wash-in and eventually, in the presence of full steady state NS309 concentration, the cell became silent when hyperpolarised below the AP threshold. During wash-out of NS309, this cell initially depolarised to the AP threshold level and fired full APs. The cell then returned to the baseline and displayed DLAMOs (see enlarged epochs in grey rectangles). *D*, the NS309-induced hyperpolarisations persisted in the presence of 1 *μ*m TTX which blocks AP-dependent synaptic communication, suggesting a direct effect of NS309 on the cells (*n* = 5). In *D*, two traces are shown from the same cell. *E*, overall, NS309 (20 *μ*m) significantly hyperpolarised all the MHb neurons tested by 14.9 ± 2.8 mV. This suggests that reduction in small conductance Ca^2+^-dependent K^+^ currents contributes to the depolarised MHb cell states. Scale bars in *A* and *B* are 200 *μ*m. 3V, third ventricle. Paired *t* test, **P < 0.01.

### Electrical states of MHb neurones are intrinsically generated

To test whether the four states of MHb neurones described above are generated intrinsically, recordings were made from 43 MHb neurones before and following blockade of AP-dependent synaptic communication through bath application of TTX. In the presence of 1 *μ*m TTX, most MHb neurones (*n* = 24/43; 56%) were hyperpolarised (mean: −3.5 ± 0.3 mV) (Fig. 6*A*–*D*), while a few cells (*n* = 9/43; 21%) were depolarised (mean: 5.7 ± 1.8 mV; data not shown). Overall, in these cells, TTX did not significantly alter RMP (*P* > 0.05; paired *t* test). The remaining 10 cells (23%) did not change their RMP in response to TTX application. This indicates that while these cells receive inhibitory and/or excitatory inputs, the acute influences of these afferent signals on the cells’ intrinsic states were comparatively small.

**Figure 6:**
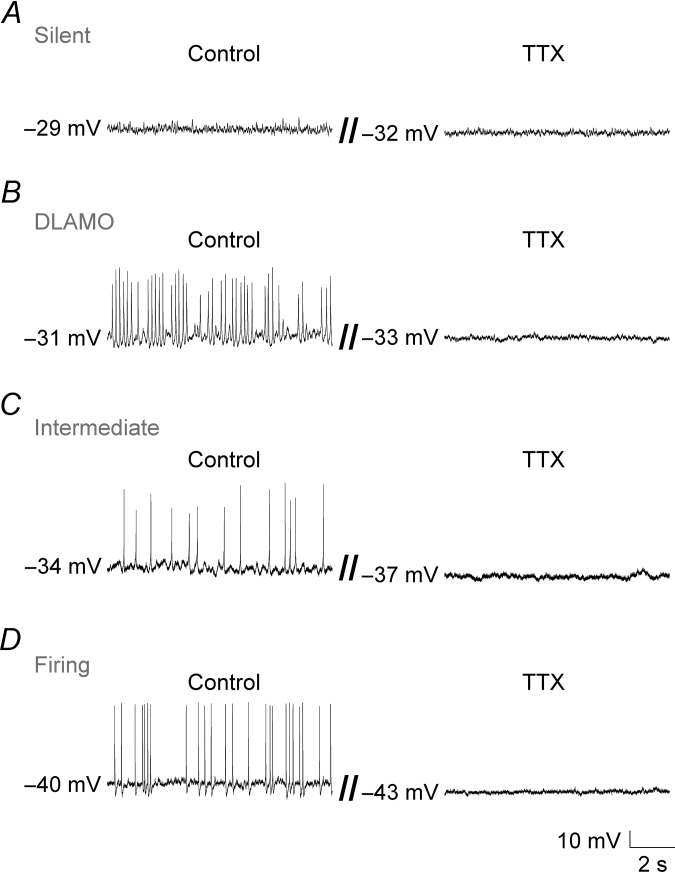
The majority of MHb neurones (*n* = 24/43; 56%), resting either at silent (*A*), DLAMO (*B*), intermediate (*C*) or firing (*D*) state, were hyperpolarised after tetrodotoxin (TTX) application. The RMPs during TTX application were not significantly different to controls, indicating that in MHb neurones RMP is not overtly determined by extrinsic synaptic signals.

### Circadian variation in the membrane properties of MHb neurones

Since MHb neurones show electrical states similar to neurones in the master circadian clock in the SCN, and because the SCN neurones alter their electrical state across the day–night cycle, we next tested whether the membrane properties of MHb neurones also vary across the projected LD cycle. To do this we recorded the basic properties (RMP, *R*_input_ and firing rate) of 230 MHb neurones and analysed them in 4 h time bins (ZT 0–4, 4–8, 8–12, 12–16, 16–20, and 20–24), with the data for ZT 8–12 coming from the recordings of 48 randomly selected cells from the above-described sample of 93 cells. Data in each 4 h time bin came from an average of ∼38 cells, ranging between 29 and 48 cells.

The RMP values of all the four different states of MHb neurones were separately plotted *versus* ZT time (Fig. 7*A*), and we observed the silent MHb neurones only from the late subjective day (∼ZT 7.8) to the late subjective night (∼ZT 20.8). This state was not recorded during other times of the day. Similarly, the depolarised cells displaying DLAMOs were more readily detected from late day (ZT 7) to late night (ZT 24), but were infrequent or absent in the morning (ZT 0–6). Consequently, cells in either the firing or intermediate states were more readily detected in the morning, although they were found infrequently throughout the day–night cycle. Figure 7*B* shows that the proportions of MHb neurones in the four cell states in each 4 h time bin varied significantly across the projected day–night cycle (*P* < 0.05; *χ*^2^ test). Interestingly, since a high number of cells were resting at depolarised states between ZT 8 and 20, we observed significant variation in RMP across the 4 h time bins (one-way ANOVA, *P* < 0.05), with average RMP being most depolarised at ZT 12–16 (−33.5 ± 0.8 mV) which was significantly higher than the average RMP recorded at ZT 20–24 (−37.3 ± 0.8 mV; Tukey's *post hoc* test, *P* < 0.05; Fig. 7*C*). These results demonstrate that the RMP of the MHb neurones varies across the projected day–night cycle with more depolarised cell states found around late day/early evening.

**Figure 7:**
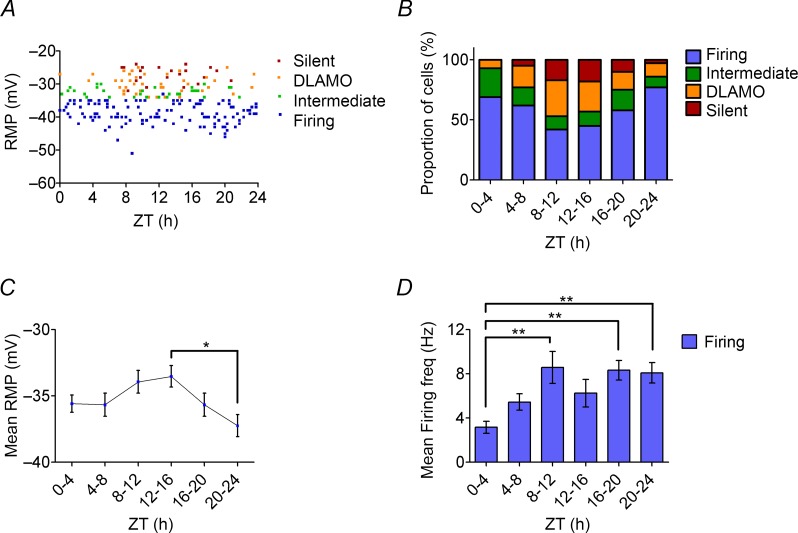
*A*, scatter plot showing the RMP of the different spontaneous states of MHb neurones *versus* ZT time, with each data point representing an individual cell. *B*, the proportions of the cells in the different spontaneous states in each 4 h time bin are shown as percentages; these varied significantly across the 4 h time bins (*P* < 0.05; *χ*^2^ test). *C*, the mean RMP values in 4 h time bins of the MHb neurones varied across the projected day–night cycle and were significantly higher during the early night (ZT 12–16; −33.5 ± 0.8 mV), but were at their lowest at late night (ZT 20–24; −37.3 ± 0.8 mV). *D*, the mean firing rate in 4 h time bins of the MHb neurones showed significant variation in the firing rate across the projected day–night cycle (one-way ANOVA, *P* < 0.001), with maximal firing rates occurring at late day (ZT 8–12; 8.6 ± 1.9 Hz) and later in the night ZT 16–20; 8.3 ± 0.9 Hz), while minimal firing rates occurred in the early day (ZT 0–4; 3.2 ± 0.5 Hz). Tukey's *post hoc* test; *P < 0.05, **P < 0.01.

To examine this more closely, we further analysed 132 of the 230 MHb neurones (57%) recorded across the projected day–night cycle that were in the spontaneous firing state, discharging full APs across a broad range of frequencies (0.2–24 Hz). Data were grouped into 4 h bins with a mean of 22 cells per time bin (range 18–27 cells). The firing rate varied significantly across the projected day–night cycle (one-way ANOVA, *P* < 0.01) rising from the nadir of 3.2 ± 0.5 Hz at early morning (ZT 0–4) to significantly higher levels of 8.6 ± 1.5 Hz at late day (ZT 8–12) and middle to late night (8.3 ± 0.9 Hz; ZT 16–20 and 20–24; Tukey's *post hoc* test, all *P* < 0.01; Fig. 7*D*). Finally, we also investigated whether the *R*_input_ of MHb neurones shows any circadian-related changes, and no significant time-dependent differences were found (one-way ANOVA, *P* > 0.05; data not shown). Taken together, these results suggest that some properties of the spontaneous neural activity of MHb neurones express circadian rhythmicity.

### Circadian variation in MHb neuronal activity depends on the molecular clock

As previously reported, circadian oscillations in PER2::Luc bioluminescence are observed in the mouse habenular complex, and unlike the SCN where these rhythms persist for up to 2 weeks, in the habenula they damp rapidly within 1–3 days (Guilding *et al*. 2010). In the current study, we assessed the circadian characteristics of the habenula in *Cry1^+/+^Cry2^+/+^* animals which have a fully functioning molecular clock, and *Cry1*^−/−^*Cry2*^−/−^ mice, in which the conventional molecular circadian clock does not function. For this, we performed long-term luminometry of *Per1*-luc expression recorded in photomultiplier tubes for up to 5 days. Bioluminescence recordings of *Cry1^+/+^Cry2^+/+^* habenula slices revealed circadian oscillations with an average period of 22.9 ± 1.9 h and a rate of damping of 3.4 ± 0.6 days (Fig. 8*A*). No rhythms were detected in *Cry1*^−/−^*Cry2*^−/−^ habenula cultures (Fig. 8*B*), and therefore rhythm parameters could not be analysed. These demonstrate that the habenula possesses a degree of intrinsic oscillatory capability that is dependent on the conventional molecular clock.

**Figure 8:**
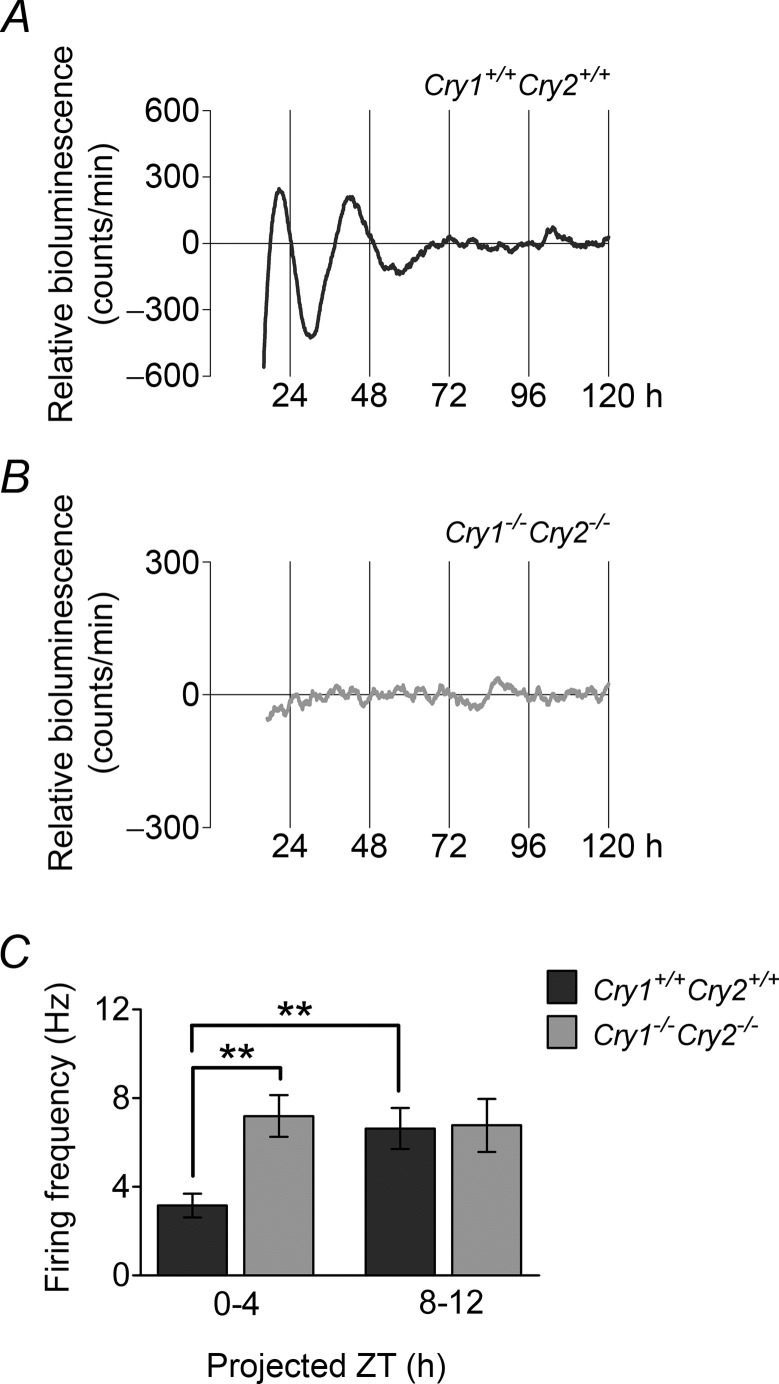
Representative detrended *Per1*-luc bioluminescence rhythm from *Cry1^+/+^Cry2^+/+^* (*A*) and *Cry1^−/−^Cry2*^−/−^ (*B*) habenular complex over 5 days in culture. *C*, the mean firing rate recorded in MHb neurones from *Cry1^+/+^Cry2^+/+^* mice was significantly lower in the morning (projected ZT 0–4; 2.9 ± 0.5 Hz) compared to projected ZT 8–12 (6.6 ± 0.9 Hz). The mean firing rate of MHb neurones from *Cry1^−/−^Cry2*^−/−^ mice, however, did not significantly differ between projected ZT 0–4 (7.2 ± 0.9 Hz) and projected ZT 8–12 (6.7 ± 1.2 Hz). Two-way ANOVA with *a priori* single degree of freedom tests; **P < 0.01.

Since the above data indicate circadian variation in the bioelectrical properties of MHb neurones, we next tested whether the absence of the intracellular molecular clock and hence the absence of overt circadian influences would affect the expression of these properties. To do this, we made recordings in MHb brain slices prepared from *Cry1*^−/−^*Cry2*^−/−^ mice, and their congenic *Cry1^+/+^Cry2^+/+^* littermates. We targeted our recordings at two epochs corresponding to projected ZT 0–4 and 8–12, since these corresponded to phases of the LD cycle at which the spontaneous firing rate of *Per1*::d2EGFP mouse MHb neurones showed significant changes.

Sixty-one *Cry1^+/+^Cry2^+/+^* MHb neurones were recorded and subjected to depolarising and hyperpolarising current injections as described above. Thirty-seven of these 61 neurones (61%) were spontaneously firing APs, whereas the remaining cells (*n* = 24/61; 29%) showed either silent, DLAMO or intermediate behaviour. These cells showed similar responses to hyperpolarising and depolarising current injections as *Per1*::EGFP MHb neurones (data not shown). Fifty-four MHb cells were recorded from *Cry1*^−/−^*Cry2*^−/−^ mice and most of these were spontaneously discharging APs (31/54; 57%). These neurones showed very similar responses to depolarising and hyperpolarising pulses to those of *Cry1^+/+^Cry2^+/+^* MHb neurones, indicating that the basic properties of MHb neurones were not overtly altered by the absence of the *Cry* genes. Further, when the firing rate of MHb neurones from these two gentoypes were compared in two 4 h time bins corresponding to projected ZT 0–4 and 8–12, two-way ANOVA indicated a significant effect of genotype (*F*_(1,63)_ = 5.8; *P *< 0.05) and time × genotype interaction (*F*_(1,63)_ = 5.1; *P *< 0.05), with the effect of time approaching significance (*F*_(1,63)_ = 3.2; *P* = 0.08). An *a priori* single degree of freedom test indicated that the firing rate of the MHb neurones recorded from congenic *Cry1^+/+^Cry2^+/+^* mice also varied significantly between the two epochs with low firing observed at projected ZT 0–4 (mean: 2.9 ± 0.5 Hz) and higher firing seen at projected ZT 8–12 (mean: 6.6 ± 0.9 Hz; *P* < 0.01; Fig. 8*C*). However, the firing rate of MHb neurones from *Cry1*^−/−^*Cry2*^−/−^ mice did not vary between these time periods (*a priori* single degree of freedom test, *P* > 0.05). Indeed, the mean firing rate in the morning (7.2 ± 0.9 Hz) was much higher than that of the *Cry1^+/+^Cry2^+/+^* MHb neurones recorded at this phase (*a priori* single degree of test, *P* < 0.01; Fig. 8*C*). These results demonstrate that the lack of the intracellular circadian clock does not influence the basic electrophysiological properties of mouse MHb neurones, but does alter the temporal variation in their spontaneous firing rate.

## Discussion

Here we demonstrate that mouse MHb neurones exhibit unusual electrophysiological properties as well as circadian variation in their RMP and firing rate characteristics. Notably, mouse MHb neurones show depolarised non-firing states that have not been reported in this structure. Most MHb neurones can, depending upon their RMP, switch between non-firing and AP firing states, establishing that such patterns of activity represent the range of possible bioelectrical states of MHb cells. Indeed, these states are intrinsic properties of most MHb neurones since they persist in the blockade of synaptic input. Alterations in SK channel activity contribute to the ionic mechanisms of these states. The circadian variation in the expression of these properties depends on a functioning conventional molecular circadian clock as they are absent in MHb brain slices prepared from *Cry1^−/−^Cry2^−/−^* mice. In other brain areas (Granados-Fuentes *et al*. 2004; Guilding *et al*. 2009), extracellular recordings reveal rhythms in neuronal activity and here we show for the first time how circadian processes can influence membrane properties of neurones in extra-SCN brain sites. Therefore, mouse MHb neurones exhibit unexpected and hitherto unreported bioelectrical activity, potentially indicating more complex roles for the MHb in neural processes and control of behaviour.

Our finding that mouse MHb neurones show circadian variation in firing rate builds on an earlier report on rat MHb neurones (Zhao & Rusak, 2005). In that study, extracellular recordings were made from spontaneously discharging (typically 3–6 spikes s^−l^) rat MHb neurones in brain slices and although they found no obvious peak or trough in spike production across the circadian cycle, they did show that rat MHb neuronal firing is typically at its lowest (∼3.2 spikes s^−l^) early in the morning (ZT 0–2) and increases to reach mean maximal levels (∼4.8–5.2 spikes s^−l^) at a number of points in the circadian cycle (ZT 6, 10 and 20) (firing rates estimated from Fig. 7 of Zhao & Rusak, 2005). In our study we also found similar firing rates (0.2–24 spikes s^−l^; mean firing rate of ∼7.6 spikes s^−l^) as did another patch-clamp investigation of rat MHb neurones (0.5–12 spikes s^−l^; mean firing rate of ∼5 spikes s^−l^; Kim & Chung, 2007). Since measurement of spontaneous firing rate in rodent MHb neurones is not overtly influenced by recording configuration and because the MHb neuronal AP discharge peaks over several hours (ZT 6–16), this raises the possibility that different MHb neuronal states were differentially sampled by the different approaches used in the two studies. Indeed, some of the depolarised states we recorded in whole-cell current clamp mode will not be detectable by extracellular configuration.

In the SCN, temporal changes in neuronal firing rate are also accompanied by daily alteration in RMP (Kuhlman & McMahon, 2004; Belle *et al*. 2009). Such change in RMP was also seen for MHb neurones and we note distinct temporal variation in our detection of the highly depolarised non-firing states of MHb neurones. All MHb neurones in this silent state were detected in the late day and night (∼ZT 8–20) and were absent in the very late night and morning (∼ZT 20–8). Analysis of the RMP of all cells combined indicates a clear and significant variation in RMP across the projected day–night cycle. Higher mean RMP (∼ −33 mV) is observed during the late day to early evening (ZT 8–16), while lower mean RMP (∼ −37 mV) is found in the very late night (ZT 20–24). Hence, circadian variation in RMP was also present in the mouse MHb.

The RMP values for mouse MHb neurones measured here are more depolarised than those reported for rat MHb neurones (Kim & Chang, 2005; Kim & Chung, 2007). These studies do not report silent cells and describe only firing activity in rat MHb neurones with RMPs ranging from −50 to −70 mV. These inter-experimental differences are most likely attributable to the age and species of rodent used. Kim and colleagues (2005, 2007) obtained MHb slices from infant and pre-weaned rats (8–24 days of age), whereas we used weanling and young adult mice. Throughout the rodent central nervous system, there are well-documented developmental changes in the electrophysiological characteristics of neurones such as cerebellar Purkinje cells (McKay & Turner, 2004) and parafascicular cells (Phelan *et al*. 2005), as well as ontogenetic alterations in ion channel expression (Gymnopoulos *et al*. 2013). So, differences in the age of the animals used are likely to contribute to inter-study differences in the electrophysiological states of the MHb neurones. Variation due to species differences is also a likely contributor. For example, in the SCN, recent research on mouse SCN neurones reports daytime depolarised RMPs of ∼ −25 to −40 mV (Kuhlman & McMahon, 2004; Belle *et al*. 2009), whereas earlier studies on rat SCN neurones reported values of ∼ −55 to −65 mV (Wheal & Thomson, 1984; Pennartz *et al*. 1998). Therefore, ontogenetic and species differences are the most likely sources of the inter-experiment reports of rodent MHb neuronal state.

Based on neuronal morphology, the rat MHb has been organised into five subnuclei (Andres *et al*. 1999), but electrophysiological studies have not revealed subnucleus-related differences in the bioelectric characteristics of MHb neurones (Kim & Chang, 2005; Kim & Chung, 2007). Indeed, in our study, we recorded from throughout the MHb and did not detect any obvious electrophysiological characteristics specific to cells in any subnucleus. This suggests that neurones throughout all subnuclei of the MHb are under some degree of circadian influence.

In the present investigation, we also observed the patterns of responses of MHb neurones to brief excitatory depolarising and inhibitory hyperpolarising current injections. Of note is that the depolarised silent, DLAMO and mixed cells produce an obvious AHP when a brief negative current is injected, and during this AHP they can briefly produce APs. This suggests that the MHb neurones that are spontaneously resting at a depolarised state should fire APs upon receiving transient inhibitory inputs, such as those arriving from the medial septum or nucleus of the diagonal band of Broca (Qin & Luo, 2009).

Consistent with previous reports (Stocker & Pedarzani, 2000; Tacconi *et al*. 2001), SK channel expression was dense in the MHb and indeed all depolarised cells tested (8 of 8) were hyperpolarised by a pharmacological activator of SK channels, NS309 (Strobaek *et al*. 2004). This indicates that SK channel activity influences the RMP of MHb neurones. Interestingly, the input resistance of MHb neurones did not vary significantly across the different neuronal states (*R*_input_ = 2.0–2.4 GΩ). This indicates that in addition to SK channel activity, multiple depolarising and hyperpolarising conductances contribute to the different states of MHb neurones.

Clock gene expression in the MHb has not been thoroughly described, although *in situ* hybridisation studies report *Per1* expression in the hamster MHb (Yamamoto *et al*. 2001) and that *Clock*, *Per1* and *Per2* are expressed in the rat MHb (Shieh, 2003). Unfortunately, *Per1*-driven EGFP was difficult to visualise in mouse MHb neurones, but this could be due to the short promotor sequence of the reporter construct as well as the lower level of expression of clock genes in the MHb compared with the SCN (Yamamoto *et al*. 2001; Shieh, 2003). Additionally, since in the SCN the degree of synchrony in neuronal activity influences the amplitude of the molecular oscillator (Brown & Piggins, 2007), the very low level of expression of *Per1*-driven EGFP may reflect reduced MHb cellular synchrony. Indeed, compared to studies on the SCN, the profile of firing rate of MHb neurones has a much broader peak, suggesting less synchronisation in the electrical activity of MHb neurones. Further, while our *Per1*-luc recordings show rhythms in *Per1*-driven bioluminescence in Hb slices that do not contain the SCN, these rhythms damp relatively quickly. This is concordant with our earlier findings with measurement of PER2::Luc rhythms (Guilding *et al*. 2010), which collectively establish the presence of some degree of intrinsic oscillatory capability in the Hb, but also indicate that cells here do not maintain synchronised circadian activities. Rhythms in *Per1*-driven bioluminescence are absent in Hb slices from *Cry1^−/−^Cry2^−/−^* mice which lack the molecular clock, indicating that these *ex vivo* clock gene rhythms are dependent on a functional molecular clock. These observations are consistent with our other studies showing that PER2::Luc rhythms in Hb tissues slices are predictably affected by mutations in the intracellular molecular oscillator (Guilding *et al*. 2013), and are concordant with our finding that morning to late day changes in electrophysiological activity are not detected in the *Cry1^−/−^Cry2^−/−^* MHb.

The functions of the MHb in the circadian timekeeping are unknown, although the adjacent LHb is implicated in the expression of circadian rhythms in behaviour (Paul *et al*. 2011) and expresses melatonin binding sites (Weaver *et al*. 1989). Indeed, based on previous electrophysiological (Zhao & Rusak, 2005) and bioluminescence imaging (Guilding *et al*. 2010), the LHb shows more overt circadian rhythmicity than the MHb and since some MHb neuronal outputs terminate in the LHb, the MHb may act to influence the circadian output of the LHb. The Hb is innervated by melanopsin-expressing retinal ganglion cells (Hattar *et al*. 2006) and both MHb and LHb neurones respond to retinal illumination (Zhao & Rusak, 2005). This suggests that these epithalamic structures have access to and respond to similar photic information that synchronises the SCN clock to the exogenous light–dark cycle. Intriguingly, the SCN communicates with the rest of the brain via paracrine factors (Silver *et al*. 1996) and may also directly innervate the habenula (Zhang *et al*. 2009). Indeed, similar to the SCN, MHb neurones are less active in the morning than at other times of the day and are more hyperpolarised at night. This indicates potential temporal coordination between these two structures. Therefore, local and/or SCN-derived circadian as well as photic cues can regulate the MHb, and subsequently may influence neuronal activity in the key target of MHb neurones, the interpeduncular nucleus (IPN). Through the IPN, the MHb could regulate the sleep–wake cycle (Haun *et al*. 1992, Valjakka *et al*. 1998) as well as the timing of responses to substances of abuse, such as nicotine (Baldwin *et al*. 2011; Fowler *et al*. 2011). Further, the triangular septum–MHb–IPN pathway is implicated in the regulation of fear and anxiety (Yamaguchi *et al*. 2013), and the MHb may then act to influence day–night activation of these brain and behaviour states.

Collectively the results of this study demonstrate both unusual states of MHb neurones as well as circadian variation in firing rate and RMP. Moreover, the daily timing of MHb neuronal states depends on the functioning of a conventional molecular clock. This study raises the possibility that the MHb functions as slave oscillator in the mammalian brain. These data indicate that MHb regulation of brain and behaviour is more complex than suggested by previous work and firmly establish that time of day must be accounted for when exploring the functions of the MHb.

## Key points

- Neurones of the suprachiasmatic nucleus (SCN) contain a molecular clock that drives these cells to exhibit daily rhythms in electrical activity.
- The molecular clock may also be present in another brain structure, the medial habenula, and here we tested whether medial habenula neurones show daily changes in their electrical activity.
- Using a brain slice preparation in which the medial habenula is isolated from inputs from the SCN, we made recordings from mouse medial habenula neurones and determined that they exhibit daily variation in their electrical properties.
- By contrast, in mice lacking functional molecular clocks, medial habenula neurones did not show overt daily change in their electrical activity.
- These studies indicate for the first time that medial habenula neurones exhibit daily changes in electrical activity that require a functional molecular clock, but do not depend on signals from the SCN.

## Abbreviations

aCSF: artificial cerebrospinal fluid
AHP: after-hyperpolarisation
AP: action potential
*Cry*/CRY: cryptochrome gene/protein
DLAMO: depolarised low-amplitude membrane oscillations
EGFP: enhanced destabilised green fluorescent protein
Hb: habenula
IPN: interpeduncular nucleus
LD: light–dark cycle
LHb: lateral habenula
Luc: luciferase
MHb: medial habenula
*Per*/PER: period gene/protein
*Per1*::GFP: *Per1*::d2EGFP transgene
PER2::Luc: *mPER2^Luc^* fusion protein
*R*_input_: input resistance
RMP: resting membrane potential
SCN: suprachiasmatic nucleus
SK: small-conductance calcium-activated potassium channel
SK3: small-conductance calcium-activated potassium channel 3
ZT: Zeitgeber time
