# Supplementary material for: Daily variation in the electrophysiological activity of mouse medial habenula neurones
Source: J Physiol. 2013 Dec 16;592(Pt 4):587–603. doi: 10.1113/jphysiol.2013.263319 (PMC3934703; doi:10.1113/jphysiol.2013.263319)
Supplement: Supplementary file 1 [file tjp0592-0587-sd1.zip › tjp5983-sup-0001-text_legends1&2.docx]

**Supplemental Fig. S1**

Quantification of membrane properties of MHb neurones recorded in whole-cell current clamp mode. (**A**) The spike amplitude was measured with respect to RMP (lower dotted line), whereas the spike width was taken at half-amplitude. The spike threshold level is marked by the upper dotted line (Pennartz et al. 1998). (**B**) To calculate the Rinput by using Ohms Law, a negative current was injected (–20pA: 500ms) and difference between the most negative voltage reached during the current pulse (V(n)) and baseline value (V(m)) measured. (**C**) In 33cells resting at depolarised membrane values, the release of negative current injection triggered an AHP that was accompanied by a train of APs. The AHP amplitude was quantified as the difference between the baseline value and the most negative voltage reached during the AHP. Duration of the AHP was taken from the onset of the AHP to the time when the original RMP value is reached.

**Supplemental Fig. S2**

*Per1***::**EGFP expression in living mouse brain slices. **A)** Illustrates the expression of

*Per1*::EGFP in the suprachiasmatic nucleus (SCN), with the inset showing the typical bright

cells seen during the late day (ZT8). **B)** Shows *Per1*::EGFP in medial habenula (MHb) with

the inset depicting the faint cells found at this time (ZT8) in this structure. Photomicrographs

of a coronal brain section showing the SCN **(A)** and MHb **(B)** taken with a 4x objective lens,

while insets were taken with a 40x objective lens. These images were taken with similar

camera settings (exposure time: 170ms; gain: 8.4x).
